# Affinity kinetics of leptin-reactive immunoglobulins are associated with plasma leptin and markers of obesity and diabetes

**DOI:** 10.1038/s41387-018-0044-y

**Published:** 2018-05-24

**Authors:** Houda Bouhajja, Noura Bougacha-Elleuch, Nicolas Lucas, Romain Legrand, Rim Marrakchi, Srini V. Kaveri, Kamel Jamoussi, Hammadi Ayadi, Mohamed Abid, Mouna Mnif-Feki, Sergueï O. Fetissov

**Affiliations:** 1 Unit of Obesity and Metabolic Syndrome, Department of Endocrinology, Hedi Chaker Hospital, Sfax, Tunisia; 2 Laboratory of Molecular and Functional Genetics, Faculty of Sciences of Sfax, University of Sfax, Sfax, Tunisia; 3 TargEDys SA, Rouen, France; 4 Biochemistry Laboratory, Hedi Chaker Hospital, Sfax, Tunisia; 5 Inserm UMRS 1138, Centre de Recherche des Cordeliers, Université Paris Descartes, Paris, France; 6 Laboratory of Molecular and Cellular Screening Processes, Centre of Biotechnology of Sfax, Sfax, Tunisia; 7 Nutrition, Gut and Brain Laboratory, Inserm UMR1073, Rouen, France; 8 Laboratory of Neuronal and Neuroendocrine Differentiation and Communication, Inserm UMR1239, Mont-Saint-Aignan, France; 9 University of Rouen Normandy, Institute for Research and Innovation in Biomedicine (IRIB), Rouen, France

## Abstract

Obese subjects display elevated plasma levels of leptin reflecting the phenomenon of leptin resistance. Here, we aimed to determine whether leptin-reactive immunoglobulins (Ig) are present in obese and type 2 diabetes (T2D) patients and whether their plasma levels and affinity kinetics may correlate with obesity and diabetes markers. We show that leptin levels are increased in obese patients with and without T2D. Although mean plasma levels of leptin-reactive IgG were similar between study groups, IgG in obese non-diabetic patients had increased dissociation rate and lower affinity (increased dissociation equilibrium constant value; KD). In controls and diabetic patients, the association rates of leptin IgG correlated negatively with obesity and diabetes markers, respectively. In contrast, KD values correlated positively with plasma leptin levels and obesity traits in our cohort, and with diabetes markers in both the total cohort and in the obese T2D group. Taken together, our data reveal that leptin-reactive IgG are present in healthy subjects, obese, and diabetic patients but display altered affinity kinetics in obesity. Increased IgG binding to leptin in healthy subjects associated with lower body mass index (BMI) suggests an enhancing role of IgG in leptin signaling. Accordingly, a decreased affinity of IgG for leptin, found in obese patients, can be relevant to leptin resistance.

## Introduction

The protein hormone leptin plays a major role in regulation of energy metabolism with pronounced anorexigenic and antidiabetic effects^1^. Adipose tissue expression and plasma leptin levels are elevated in obesity, leading to the concept of functional leptin resistance, but its mechanism remains unknown^2–5^. It has been found that the majority of leptin circulates in a bound form with several serum/plasma proteins. However, in obesity higher levels of free non-bound leptin was present, pointing to the relevance of leptin binding proteins to leptin resistance^6,7^. Soluble leptin receptor and C-reactive protein (CRP) were the first leptin binding proteins identified^8,9^. The molecular weight of some leptin binding proteins reported in these studies indicates that they may include immunoglobulins (Igs). Indeed, the presence of leptin-reactive IgG in healthy subjects and in rats has previously been demonstrated^10^. Importantly, IgG are different from other hormone-binding proteins because of their variable molecular structure in the Fab region, underlying different kinetics of interaction with the ligand. This implies that natural leptin-reactive IgG autoantibodies (autoAbs) may modulate the biological activity of leptin depending on their IgG binding properties. However, the presence and properties of leptin-reactive IgG have not been studied in obesity and diabetes. Thus, in this study, we analyzed plasma samples from healthy subjects and patients with obesity and/or type 2 diabetes (T2D) to characterize circulating IgG autoAbs reactive with leptin. We measured affinity kinetics between plasma extracted IgG and leptin, and further evaluated their potential link with obesity and diabetes using a statistical correlation analysis.

## Material and methods

The total cohort included 20 obese, 28 obese T2D (Ob T2D), 30 non-obese T2D (lean T2D) patients, and 30 healthy study participants (controls) that were admitted to the department of endocrinology at the Hospital Hedi Chaker (Sfax, Tunisia). The detailed patient recruitment procedure has been described elsewhere^11^. Obesity and T2D were diagnosed according to the World Health Organization (WHO) criteria^12,13^. Diabetic patients were slightly older than obese and controls (Table S1). Venous blood samples were collected from each participant after fasting overnight. All patients were informed of the nature of the study. Plasma levels of IgG autoAbs reacting with human recombinant leptin (Sigma, St. Louis, MO, USA) were measured using ELISA^14^. Total IgG were purified from plasma samples using the Melon Gel Kit (ThermoFischer Scientic, Rockford, IL, USA). Affinity kinetics of purified IgG for leptin was determined by surface plasmon resonance (SPR) on a BIAcore 1000 instrument (GE Healthcare) as previously published^15^. Human recombinant leptin (Sigma, St. Louis, MO, USA) was covalently coupled on a CM5 chip (GE Healthcare) using the amine coupling kit (GE Healthcare) resulting in immobilized leptin in the amount of 2000 resonance units (RU). The affinity kinetic data were analyzed using BiaEvaluation 4.1.1 software (GE Healthcare). For fitting of kinetic data, the Langmuir’s 1:1 model was used and the sample values were corrected by subtracting the blank values resulting from the injection of HBS-EP buffer. The association rate (Ka), the dissociation rate (Kd) and the dissociation equilibrium constant (KD) were obtained by the analysis of the fitted sensorgrams. Data were analyzed and the graphs were plotted using GraphPad Prism 6.0 (GraphPad Software Inc., San Diego, CA). Normality was evaluated by Shapiro-Wilk test. Data are presented as median or mean according to the normality of variables. Outliers have been identified based on ROUT method (Robust regression and Outlier removal). Intergroup comparisons were performed using the nonparametric analysis of variance (ANOVA) Kruskal–Wallis (K–W) followed by Dunn’s multiple comparisons post-hoc test. Individual groups were compared using Mann–Whitney test. Spearman’s correlation (rho) was performed to evaluate potential associations of levels and affinity kinetics of leptin-reactive IgG with clinical markers of obesity and diabetes. A *p*-value < 0.05 was considered significant.

## Results

**Fig. 1:**
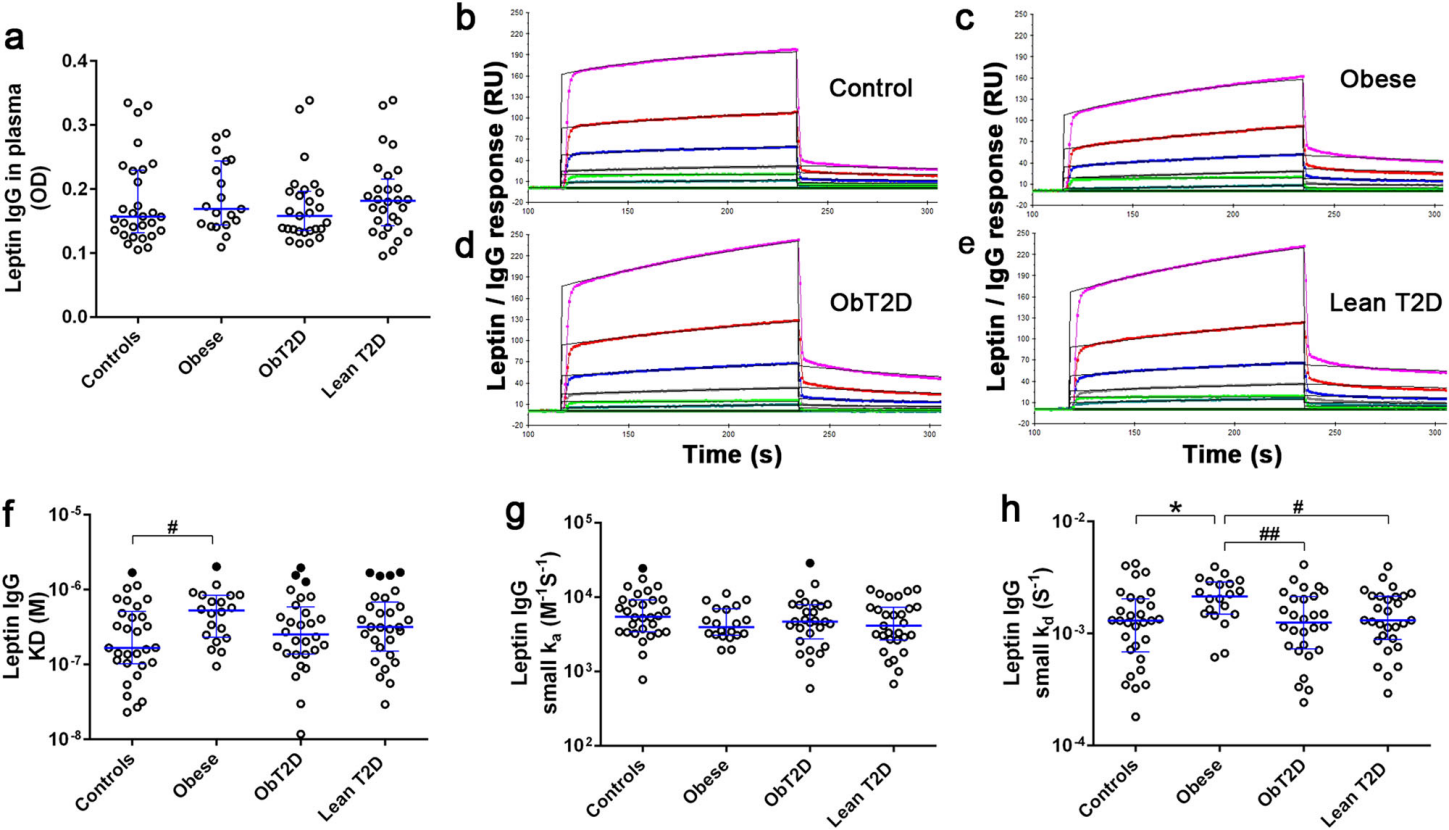
Properties of leptin-reactive IgG. Plasma levels of leptin-reactive IgG (**a**). Representative examples of curve fits for the affinity kinetic analysis of a control subject (**b**), obese (**c**), obese T2D (**d**) and lean T2D (**e**) patients. Affinity kinetics between human leptin and IgG showing: dissociation equilibrium constants (KD, **f**), association rates (small Ka, **g**) and dissociation rates (small Kd, **h**). The data shown are median ± interquartile range values. **f** K–W test, *p* = 0.084; M–W test, ♯*p* < 0.05 obese vs. controls. **h** K–W test, *p* = 0.026; Dunn’s *p < 0.05 obese vs. controls; M–W test, ♯*p* < 0.05 obese vs. lean T2D, ♯♯*p* < 0.01 obese vs.ObT2D. Filled circles indicate values statistically identified as outliers. (Controls, *n* = 30; Obese, *n* = 20; ObT2D, *n* = 28 and lean T2D, *n* = 30, error bars: interquartile range).ObT2D, Obese T2D; T2D, type 2 diabetes

Clinical characteristics of the study groups are shown in Table S1. As expected, leptin levels were increased in obese patients with and without T2D as compared to controls and lean T2D groups (Table S1). Leptin-reactive IgG were detected at variable levels in plasma of all study subjects without significant differences (K–W *p* = 0.5) of their median levels between controls and patient groups (Fig. 1a).

Affinity kinetic analysis of interactions between plasma-extracted IgG and leptin using SPR (Fig. 1b–e) revealed that KD values of leptin-reactive IgG were in the micromolar range around 10^−6^ and 10^−7^ M in all study subjects (Fig. 1f). The KD values, which are an inverse measure of affinity, were significantly higher in obese subjects than in controls (by a factor of 1.6), indicating lower affinity. When values statistically identified as outliers (indicated by filled circles in Fig. 1f) were removed, significant differences between obese and other groups were found (vs. controls, KW *p* = 0.048, Dunn’s *p* < 0.05; vs. obese T2D, MW *p* = 0.016), and the difference tended to reach statistical significance vs. lean T2D group (MW *p* = 0.056). The decrease in affinity in the obese group was mainly due to higher dissociation rates (by a factor of 1.4 vs. controls, 1.5 vs. obese diabetics, and 1.4 vs. lean diabetic patients) (Fig. 1h). The IgG—leptin association rates did not differ significantly among the groups (Fig. 1g).

To further evaluate the relevance of leptin-reactive IgG to the patient phenotype, we studied whether their plasma levels and affinity kinetics correlated with clinical traits of obesity and diabetes. We found that leptin-reactive IgG levels correlated negatively with waist circumference in obese T2D group, and positively with glycated hemoglobin (HbA1c) levels in both diabetic groups (Table S2). Expectedly, plasma leptin levels correlated with anthropometric parameters in all participants and individual groups as well as with insulin levels, homeostasis model assessment of insulin resistance (HOMA-IR) and beta cell function (HOMA-β) in the total cohort (Table S2).

With regard to affinity kinetics, KD values of leptin-reactive IgG correlated positively with plasma leptin levels, body mass index (BMI), and body fat in the total cohort (Table 1). Moreover, KD values correlated positively with insulin levels and HOMA-IR index in all participants, as well as in the obese diabetic group. The association rates (small Ka) correlated negatively with obesity measures in healthy controls, but not in obese and diabetic patients. In contrast, small Ka correlated negatively with insulin levels and HOMA-IR index in diabetic patients but not in obese and controls. The dissociation rates (small Kd) correlated positively with BMI and waist circumference in the total cohort. Moreover, small Kd correlated positively with waist circumference and negatively with plasma insulin levels in obese diabetic and lean T2D patients, respectively (Table 1).

**Table 1:** The correlation coefficients, Spearman’s rho, between affinity kinetics of leptin-reactive IgG, obesity, and diabetes traits in all participants and individual groups

	All participants			Controls			Obese			Obese T2D			Lean T2D		
Variable	Small Ka	Small Kd	KD	Small Ka	Small Kd	KD	Small Ka	Small Kd	KD	Small Ka	Small Kd	KD	Small Ka	Small Kd	KD
Leptin	− 0.13	0.13	0.17*	−0.31*	0.07	0.23	0.36	**−**0.15	**−**0.25	**−**0.15	0.14	0.25	**−**0.13	**−**0.26	**−**0.05
BMI	**−**0.09	0.19*	0.17*	**−0.41** ^†^	0.15	**0.38** ^†^	**−**0.03	0.08	**−**0.00	**−**0.13	0.15	0.14	0.24	0.01	**−**0.15
WC	**−**0.08	0.19*	0.15	−0.34*	0.08	0.24	0.27	**−**0.03	**−**0.25	0.15	**0.44** ^†^	0.09	**−**0.00	0.05	0.01
Body fat	**−**0.13	0.15	0.17*	**−0.44** ^†^	**−**0.02	0.26	0.11	**−**0.00	**−**0.15	**−**0.15	0.06	0.11	**−**0.03	**−**0.01	**−**0.01
Glycemia	**−**0.15	**−**0.08	0.07	0.13	**−**0.12	**−**0.14	0.15	**−**0.27	**−**0.27	**−**0.20	0.12	0.31	**−**0.30	0.06	0.29
HbA1c	**−**0.11	**−**0.11	0.03	**−**0.12	**−**0.07	0.05	**−**0.04	0.19	0.14	0.18	**−**0.18	**−**0.23	**−**0.10	**−**0.10	**−**0.05
Insulin	**−0.25** ^††^	0.10	**0.22** ^†^	**−**0.25	0.14	0.18	0.06	**−**0.14	**−**0.25	**−0.44** ^†^	0.06	0.34*	−0.33*	−0.33*	0.02
HOMA-IR	**−0.30** ^††^	0.03	**0.23** ^†^	**−**0.24	0.13	0.19	0.00	**−**0.16	**−**0.22	**−0.44** ^†^	0.17	**0.43** ^†^	−0.36*	**−**0.27	0.07
HOMA-β	**−**0.06	0.15	0.11	**−**0.25	0.17	0.21	**−**0.09	0.13	0.07	**−**0.18	**−**0.04	0.05	**−**0.09	**−**0.22	**−**0.09

^†^*p* < 0.05; ^††^*p* < 0.01: sig (2-tailed); *p < 0.05: sig (1-tailed)

*BMI* body mass index, *WC* waist circumference, *HbA1c* glycated hemoglobin; *HOMA-IR* homeostasis model assessment of insulin resistance, *HOMA-β* Homeostasis model assessment of beta cell function

## Discussion

Our study is the first to characterize leptin-reactive IgG autoAbs in subjects with obesity and T2D. Leptin-reactive IgG and IgA have been shown to be naturally present in the plasma of healthy women^10^. Our study reveals the ubiquitous presence of leptin-reactive IgG in healthy adults of both sexes as well as in patients with obesity and T2D. Our results provide new evidence to the earlier hypothesis implicating leptin-binding proteins in obesity and the phenomenon of leptin resistance^6–8^. In fact, affinity kinetics properties of leptin-reactive IgG were found to be associated with plasma leptin levels, as well as with several anthropometric and biochemical parameters of obesity and diabetes. This indirectly suggests such autoAbs as functional leptin carrier in humans. Moreover, our finding of negative correlations between the association rates of leptin-reactive IgG and obesity markers in healthy controls, but not in obese and diabetic patients suggest a protective role of such autoAbs and their potential contribution to leptin-mediated effects. Conversely, the positive relationship between KD, dissociation rates and obesity in the total cohort suggest that a decrease in IgG affinity for leptin (increase in KD) is associated with hyperleptinemia, BMI, and obesity. Thus, altered leptin autoAbs kinetics may be relevant to the phenomenon of leptin resistance in obese patients. In summary, our findings provide new mechanistic insight on earlier data showing that obesity is accompanied by a decrease in the protein bound forms of leptin and an increase in free leptin in plasma^6–8^. Accordingly, affinity kinetics may represent a new biomarker of functionally relevant changes of leptin bioavailability, whereby IgG may act either by blocking or exposing different parts of the leptin molecule necessary for ObRb (long leptin receptor isoform) binding and activation. Previously, increased hormone protective properties of IgG associated with increased micromolar affinity have been shown for ghrelin, where they were found to enhance the hormone’s orexigenic effect^16^. Currently, the origin of different affinity kinetics of IgG for leptin or ghrelin in obesity remains unknown, and may involve stimulation by homologous antigens from gut microbiota^17^.

Given the important role of leptin in glucose metabolism^18^, the inverse relationship between association rates and affinity of leptin-reactive IgG with insulin levels and HOMA-IR index in our cohort suggests that a decrease in IgG affinity kinetics also may be related to disturbance of glucose homeostasis. Indeed, decreased affinity and IgG binding to leptin associated with hyperinsulinemia and insulin resistance were found in both groups of diabetic patients, but not in obese and healthy controls, indicating that loss of binding affinity of leptin-reactive IgG is favorable for T2D. Of relevance, acute injection of leptin-neutralizing antibodies has been shown to induce hyperinsulinemia in mice^19^.

In conclusion, we showed that IgG with micromolar affinity for leptin are naturally present in healthy subjects and in patients with obesity and T2D. Increased IgG binding rates to leptin in healthy subjects were associated with lower BMI, suggesting an enhancing role of IgG in leptin signaling. Accordingly, the decreased affinity of IgG for leptin evidenced in obese patients may play a role in leptin resistance. Further investigations need to clarify the molecular mechanisms involving leptin-reactive IgG in leptin resistance, obesity, and diabetes.

## Electronic supplementary material

Supplemental Tables 1 & 2
