# Supplementary material for: Affinity kinetics of leptin-reactive immunoglobulins are associated with plasma leptin and markers of obesity and diabetes
Source: Nutr Diabetes. 2018 May 24;8:32. doi: 10.1038/s41387-018-0044-y (PMC5966443; doi:10.1038/s41387-018-0044-y)
Supplement: Supplementary file 1 — Supplemental Tables 1 & 2 [file 41387_2018_44_MOESM1_ESM.docx]

**Supplemental data**

Table S1 : Subjects characteristics

| **Baseline characteristics** | **Controls** | **Obese** | **Obese T2D** | **Lean T2D** | ***P* value** |
| --- | --- | --- | --- | --- | --- |
| **n (Male/Female)** | 30 (18/12) | 20 (4/16) | 28 (9/19) | 30 (19/11) |  |
| **Age † (year)** | 45.47 ± 9.35 | 43.45 ± 10.62 | 57.54 ± 10.52 a,b | 57.73 ± 7.92 a,b | <0.001 |
| **Current smoking ‡, n (%)** | 12 (40) | 1 (5) | 3 (10.7) | 11 (36.7) | 0.002 |
| **Current drinking ‡, n (%)** | 2 (7.7) | 1 (5) | 0 (0) | 4 (13.3) | ns |
| **Hypertension ‡, n(%)** | 0 (0) | 3 (17.6) | 16 (57.1) | 10 (34.5) | <0.001 |
| **Family history of diabetes ‡, n (%)** | 12 (46.2) | 13 (76.5) | 24 (85.7) | 25 (83.3) | 0.003 |
| **BMI †(kg/m2)** | 23.05 (18.70-25.30) | 36 (30.1-49) a | 31.96 (29.80-40.20) a | 22.8 (17.8-25.5) b,c | <0.001 |
| **Waist circumference †(cm)** | 86.50 (73-102) | 113 (100-134) a | 114 (102-133) a | 91.5 (69-103) a,b,c | <0.001 |
| **Waist to hip ratio †** | 0.91 (0.81-1.01) | 0.95 (0.86-1.13) | 1.02 (0.82-1.85) a | 0.97 (0.81-1.11) a,c | <0.001 |
| **Body Fat † (kg)** | 14 (6.4-23.8) | 38.90 (25.3-73.4) a | 31.5 (23.6-52) a,b | 14.05 (5.1-22.4) b,c | <0.001 |
| **Leptin † (ng/ml)** | 4.76 (0.24-26.86) | 39.51 (11.83-82.31) a | 24.76 (2.10-83.20) a | 3.67 (0.53-16.29) b,c | <0.001 |
| **TC † (mmol/l)** | 4.28 ± 0.91 | 4.69 ± 0.85 | 4.73 ± 0.94 | 4.26 ± 0.92 | ns |
| **HDL-C †(mmol/l)** | 1.29 (0.79-2.12) | 1.23 (0.82-2.03) | 1.25 (0.68-2.12) | 1.17 (0.76-1.89) | ns |
| **LDL-C †(mmol/l)** | 2.60 ± 0.75 | 2.78 ± 0.65 | 2.40 ± 0.81 | 2.57 ± 0.68 | ns |
| **TG †(mmol/l)** | 0.74 (0.38-2.76) | 1.46 (0.42-3.45) a | 1.65 (0.89-4.65) a | 1.28 (0.44-3.12) a,c | <0.001 |
| **FPG †(mmol/l)** | 5 (4.4-6) | 5.4 (4.6-6.7) a | 10 (5.9-17.9) a,b | 12.1 (5.1-18.8) a,b | <0.001 |
| **2hPG †(mmol/l)** | 5.4 (3.5-7.4) | 6.5 (3.9-10) a | - | - |  |
| **HbA1c †(%)** | 5.4 (4.4-6.0) | 5.7 (4.6-6.5) a | 8.75 (6.3-15.8) a,b | 10.05 (7-17.5) a,b,c | <0.001 |
| **FINS † (μIU/ml)** | 5.28 (1.65-15.53) | 17.02 (10.08-31.07) a | 14.22 (1.60-44.40) a | 5.69 (1.26-39.71) b,c | <0.001 |
| **HOMA-IR †** | 1.15 (0.35-3.24) | 4.16 (2.42-7.18) a | 7.43 (0.57-26.84) a,b | 3.60 (0.71-17.82) a,c | <0.001 |
| **HOMA β-cell function †** | 70.34 (22.90-258.78) | 181.98 (99.30-392.04) a | 43.39 (5.32-135.82) b | 18.37 (2.77-120.32) a,b,c | <0.001 |
| **hs-CRP †(mg/l)** | 0.72 (0.17-4.74) | 4.05 (0.56-9.83) a | 2.77 (0.87-8.78) a | 1.04 (0.17-7.55) b,c | <0.001 |
| **Creatinine †(μmol/l)** | 67.28 (42.35-91.88) | 57.96 (34.83-105.74) | 61.90 (43.64-119.41) | 63.80 (38.32-102.17) | ns |
| **Uric Acid † (μmol/l)** | 242.87 (100.67-444.56) | 282.07 (204.45-488.18) a | 296.08 (110.53-541.89) | 210.5 (121.79-483.52) a,b,c | <0.001 |

Data are means ± SD, medians (min-max) for skewed variables, or numbers (percentages) for categorical variables.

†: Kruskal-Wallis test (for continuous variables); ‡ Chi-square test (for categorical variables). Test Mann-Whitney for comparison between 2 groups: a: p<0.05 vs controls; b: p<0.05 vs obese; c: p<0.05 vs obese T2D.

**BMI**: body mass index; **WC**: waist circumference; **WHR**: waist-to hip ratio; **FPG**: fasting plasma glucose; **2hPG**: 2h plasma glucose after oral glucose tolerance test; **HbA1c**: glycated hemoglobin; **FINS**: fasting plasma insulin; **HOMA-IR**: homeostasis model assessment of insulin resistance; **HOMA β-cell function**: homeostasis model assessment of beta cell function; **TC**: total cholesterol; **HDL**: high density lipoprotein; **LDL**: low density lipoprotein; **TG**: triglycerides; **hs-CRP**: highly sensitive C-reactive protein; **T2D**: type 2 diabetes.

Table S2 : The correlation coefficients, Spearman’s rho, between leptin, leptin-reactive IgG obesity and diabetes traits in all participants and individual groups

|  | **All participants** | |  | **Controls** | |  | **Obese** | |  | **Obese T2D** | |  | **Lean T2D** | |
| --- | --- | --- | --- | --- | --- | --- | --- | --- | --- | --- | --- | --- | --- | --- |
| Variable | Leptin (levels) | leptin IgG (levels) |  | Leptin (levels) | leptin IgG (levels) |  | Leptin (levels) | leptin IgG (levels) |  | Leptin (levels) | leptin IgG (levels) |  | Leptin (levels) | leptin IgG (levels) |
| Leptin | - | -0.05 |  | - | -0.03 |  | - | 0.26 |  | - | -0.25 |  | - | -0.17 |
| BMI | **0.81†††** | 0.02 |  | **0.52††** | 0.24 |  | 0.36 | 0.25 |  | **0.62†††** | -0.24 |  | **0.45†** | -0.07 |
| WC | **0.73†††** | -0.03 |  | 0.11 | 0.10 |  | 0.37 | 0.30 |  | 0.17 | **-0.34*** |  | **0.50††** | -0.18 |
| Body Fat | **0.89†††** | 0.01 |  | **0.78†††** | 0.02 |  | **0.54†** | 0.37 |  | **0.60††** | -0.30 |  | **0.80†††** | -0.14 |
|  |  |  |  |  |  |  |  |  |  |  |  |  |  |  |
| Glycemia | -0.03 | 0.02 |  | -0.25 | 0.15 |  | 0.17 | 0.12 |  | 0.07 | -0.17 |  | 0.21 | 0.08 |
| HbA1c | -0.06 | 0.10 |  | 0.25 | 0.18 |  | 0.09 | -0.21 |  | -0.15 | **0.36*** |  | -0.13 | **0.34*** |
| Insulin | **0.63†††** | 0.03 |  | 0.30 | -0.04 |  | 0.05 | -0.10 |  | 0.27 | 0.03 |  | 0.25 | 0.01 |
| HOMA-IR | **0.46†††** | 0.00 |  | 0.28 | -0.05 |  | 0.09 | -0.07 |  | 0.20 | -0.08 |  | **0.31*** | 0.09 |
| HOMA-β | **0.48†††** | 0.02 |  | **0.34*** | -0.11 |  | -0.09 | -0.17 |  | 0.13 | 0.11 |  | -0.02 | -0.13 |

† p<0.05; †† p<0.01; ††† p<0.001: sig (2-tailed)

* p<0.05: sig (1-tailed)

BMI: body mass index, WC: waist circumference, HbA1c: glycated hemoglobin; HOMA-IR: homeostasis model assessment of insulin resistance; HOMA-β: Homeostasis model assessment of beta cell function.
